# Mortality, hospitalizations, and persistence of symptoms in the outpatient setting of the first COVID-19 wave in Brazil: results of SARS-Brazil cohort study

**DOI:** 10.31744/einstein_journal/2024AO0652

**Published:** 2024-07-12

**Authors:** Henrique Andrade Rodrigues Fonseca, Adriano Jose Pereira, Ricardo Kenji Nawa, Viviane Aparecida Rodrigues Sant’Anna, Tatiana Ferreira de Almeida, Hélio Penna Guimarães, Alexandre Pereira Tognon, Lucas Miranda Marques, Lucas Santana Coelho da Silva, Rafaela de Souza Bittencourt, Camila Pachêco Gomes, Priscila de Aquino Martins, Aryadne Lyrio de Oliveira, Eveline Pipolo Milan, Frederico Toledo Campos Dall’Orto, Conrado Roberto Hoffman, Guacyra Almeida, Fábio Barlem Hohmann, Diogo Duarte Fagundes Moia, Luciana Pereira Almeida Piano, Felipe Pinheiro Machado, Ronaldo Vicente Pereira Soares, Lucas Petri Damiani, Silvia Regina Lamas Assis, Edson Amaro, Luiz Vicente Rizzo, Otávio Berwanger

**Affiliations:** 1 Hospital Israelita Albert Einstein São Paulo SP Brazil Hospital Israelita Albert Einstein, São Paulo, SP, Brazil.; 2 Hospital São Vicente de Paulo Passo Fundo RS Brazil Hospital São Vicente de Paulo, Passo Fundo, RS, Brazil; 3 Multidisciplinary Institute of Health Universidade Federal da Bahia Vitória da Conquista BA Brazil Multidisciplinary Institute of Health, Universidade Federal da Bahia, Vitória da Conquista, BA, Brazil.; 4 Department of Microbiology Universidade Estadual de Santa Cruz Ilheús BA Brazil Department of Microbiology, Universidade Estadual de Santa Cruz, Ilheús, BA, Brazil.; 5 Hospital Estadual Dr. Jayme Santos Neves Serra ES Brazil Hospital Estadual Dr. Jayme Santos Neves, Serra, ES, Brazil.; 6 Hospital Giselda Trigueiro Natal RN Brazil Hospital Giselda Trigueiro, Natal, RN, Brazil.; 7 Hospital Maternidade e Pronto Socorro Santa Lúcia Poços de Caldas MG Brazil Hospital Maternidade e Pronto Socorro Santa Lúcia, Poços de Caldas, MG, Brazil.; 8 Hospital Regional Hans Dieter Schimdt Joinville SC Brazil Hospital Regional Hans Dieter Schimdt, Joinville, SC, Brazil.; 9 Hospital de Emergência do Agreste Dr. Daniel Houly Arapiraca AL Brazil Hospital de Emergência do Agreste Dr. Daniel Houly, Arapiraca, AL, Brazil.

**Keywords:** SARS-CoV-2, COVID-19, Coronavirus infections, Mortality, Hospitalization, Long-term COVID-19 symptoms, Length of stay, Intensive care unit, Brazil

## Abstract

**Objective:**

To evaluate deaths, hospitalizations, and persistence of symptoms in patients with COVID-19 after infection in an outpatient setting during the first COVID-19 wave in Brazil.

**Methods:**

This prospective cohort was between April 2020 and February 2021. Hospitalized or non-hospitalized COVID-19 patients until five days after symptom onset were included. The outcomes measured were incidence of death, hospitalization, and persistence of more than two symptoms 60 days after discharge.

**Results:**

Out of 1,198 patients enrolled in the study, 66.7% were hospitalized. A total of 289 patients died (1 [0.3%] non-hospitalized and 288 [36%] hospitalized). At 60 days, patients non-hospitalized during admission had more persistent symptoms (16.2%) compared to hospitalized (37.1%). The COVID-19 severity variables associated with the persistence of two or more symptoms were increased age (OR= 1.03; p=0.015), respiratory rate at hospital admission (OR= 1.11; p=0.005), length of hospital stay of more than 60 days (OR= 12.24; p=0.026), and need for intensive care unit admission (OR= 2.04; p=0.038).

**Conclusion:**

COVID-19 survivors who were older, tachypneic at admission, had a hospital length of stay >60 days, and were admitted to the intensive care unit had more persistent symptoms than patients who did not require hospitalization in the early COVID-19 waves.

**ClinicalTrials.gov Identifier:** NCT04479488.

## In Brief

The SARS-Brazil study showed higher COVID-19 severity in the first wave. Higher mortality was observed during hospitalization (36% *versus* only one patient in the non-hospitalized group), particularly in aged patients needing hemodialysis and mechanical ventilation. COVID-19 survivors who were older, tachypneic at admission, had a hospital length of stay >60 days, and were admitted to the intensive care unit had more persistent symptoms than those who did not require hospitalization.

## Highlights

■ Previous influenza vaccination was associated with a reduced need for hospitalization after COVID-19 diagnosis.

■ In 60 days of outpatient setting, four deaths occurred (three in the hospitalized and one in the non-hospitalized group).

■ The persistence of more than two symptoms in 60 days was observed in 31.9% of patients.

## Graphical abstract

**Figure:**
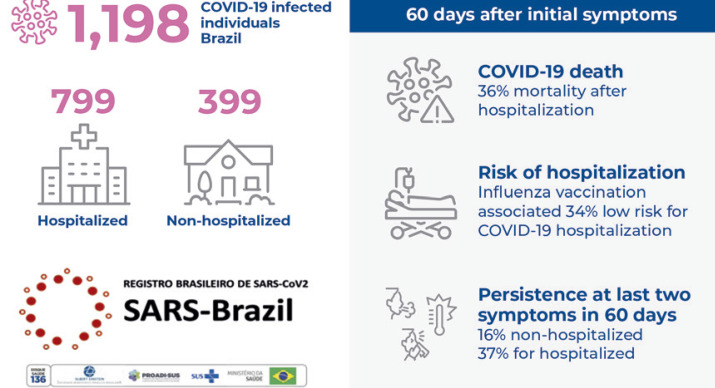

## INTRODUCTION

On March 11, 2020, the World Health Organization declared the coronavirus disease 2019 (COVID-19) a global pandemic after the first severe acute respiratory syndrome coronavirus-2 (SARS-CoV-2) infection in Wuhan, China in 2019. Primarily, COVID-19 was compared with seasonal influenza, given that both are respiratory infectious diseases presenting similar symptoms. However, the findings of the first cohort studies suggested a higher incidence of mortality and hospitalizations of COVID-19 derivatives.^(1-3)^ Data suggest that the symptoms and post-acute sequelae of SARS-CoV-2 infection can occur depending on COVID-19 severity.^(4,5)^ However, in the survivors, the long-term condition of COVID-19 is an increasing public health concern.

Hence, we performed a SARS-Brazil study to investigate whether the severity of the COVID-19 wave in Brazil is associated with the risk of death, hospitalization, and persistence of symptoms in the outpatient setting.

## OBJECTIVE

This prospective, multicenter cohort study aimed to evaluate variables associated with the predictors of mortality, hospitalization, and symptom persistence in patients with COVID-19 after the first COVID-19 cases in Brazil.

## METHODS

### Study design and setting

SARS-Brazil was a national, multicenter, prospective cohort study that included SARS-CoV-2-infected adult (≥18 years) patients from Brazil. Data were collected from 68 sites from April 2020 to February 2021 and included some of the first COVID-19 cases in Brazil.

### Patient participants

**Figure 1:**
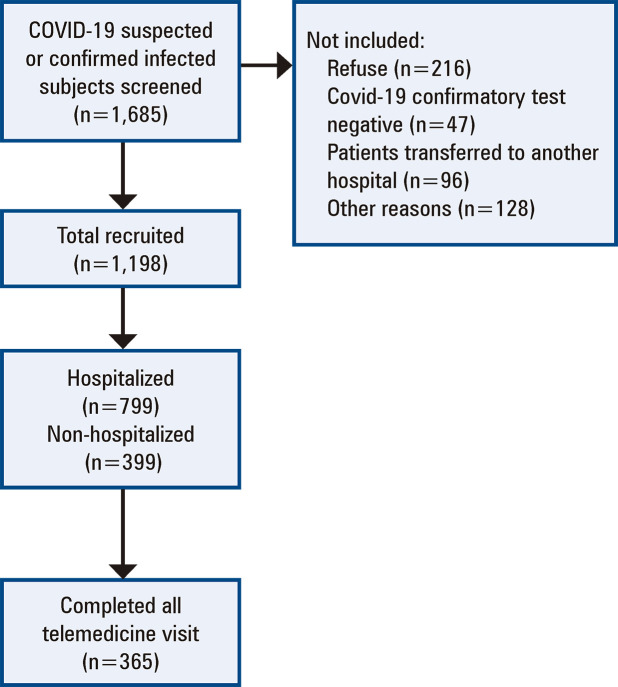
Flowchart of inclusion and exclusion of participants in the current analysis

Mild and moderate-to-severe (inpatient) individuals were prospectively included in the Brazilian SARS-CoV-2 Registry after laboratory confirmation of SARS-CoV-2 infection until five days after symptom onset. The patients were followed up for 60 days to assess the potential incidence of the study outcomes of interest. Inpatients were evaluated in the hospital and admitted to the telemedicine department after discharge. Individuals with mild COVID-19 symptoms and receiving outpatient care were automatically admitted via telemedicine for follow-up visits (Figure 1S, Supplementary Material).

### Follow-up visits

Follow-up visits were performed using a central telemedicine care system, with contact every 15 days until 60 days after discharge. In addition, telemedicine consultations were performed to determine the incidence of outcomes and medical management of health conditions of the individuals. Data were collected from a centralized clinical registry, the Research Electronic Data Capture (REDCap; Vanderbilt University, USA).

### Outcome measurement

Telemedicine visits were used to describe the incidence of dry cough, cough with phlegm, sore throat, rhinorrhea, chest pain, headache, myalgia, arthralgia, fatigue or tiredness at rest, difficulty walking, altered consciousness or mental confusion, abdominal pain, diarrhea, nausea, vomiting, other infections, the need for dialysis, bleeding, and dyspnea according to the New York Heart Association dyspnea scale.^(6)^ All deaths and hospitalizations were classified by an independent event committee.

### Statistical analyses

The study population characteristics are shown for each group (hospitalized and non-hospitalized) and the total population. Quantitative variables were described using the mean, standard deviation (SD), median, interquartile range (IQR), and minimum, maximum, and number of valid observations. Qualitative variables were presented as absolute and relative frequencies. Predictors of total mortality, hospitalization, and persistence of more than two symptoms at 60 days in an outpatient setting were investigated using univariate and multivariate logistic regression models, considering the following: age and sex, presence of chronic diseases, medications in use, and hospitalization outcomes. Patients with missing outcome data were excluded from the analysis.

Multivariable models were performed, including univariate results that showed p<0.10 in forward logistic regressions, and a plausible impact on outcomes was considered as a predictor. A variable with a p<0.05 was considered a significant factor predictor, with the outcomes at a 95% confidence level. The statistical significance level was set at a two-tailed 5%. All statistical analyses were performed using SAS, Version 9.4 (SAS Institute, Cary, NC, USA).

### Ethics

This study was approved by the research ethics committee *Hospital Israelita Albert Einstein* (CAAE: 0047620.3.1001.0071, # 4.406.928). All the study participants and/or their legal guardians provided written informed consent.

## RESULTS

### Baseline patients’ characteristics

A total of 1,685 non-consecutive patients were screened, and 1,198 were included in the cohort, while 399 were non-hospitalized, and 799 required hospitalization after initial symptoms (Figure 1). For non-hospitalized and hospitalized patients, the median [IQR] ages were 40 years [range, 32-53 years] and 60 years [range, 49-70 years], respectively. Of these, 32.8% of non-hospitalized patients and 59.6% of hospitalized patients were men (Table 1).

**Table 1:** Clinical characteristics of COVID-19 infected patients

Variables	Non-hospitalized Group (n=399)	Hospitalized Group (n=799)	Overall (n=1,198)
Age, y, median, [IQR]	40 [32 – 53.5]	60 [49 – 70]	54 [41 – 67]
Male, n (%)	131 (32.8)	476 (59.6)	607 (50.7)
Chronic conditions, n (%)			
Hypertension	71 (17.8)	467 (58.4)	538 (44.9)
Diabetes	29 (7.3)	280 (35)	309 (25.8)
Heart failure	2 (0.5)	48 (6)	50 (4.2)
Myocardial infarction	2 (0.5)	36 (4.5)	38 (3.2)
Stroke	1 (0.3)	29 (3.6)	30 (2.5)
Kidney disease	0 (0)	67 (8.4)	67 (5.6)
Asthma	26 (6.5)	53 (6.6)	79 (6.6)
COPD	5 (1.3)	42 (5.3)	47 (3.9)
Solid organ transplantation	1 (0.3)	17 (2.1)	18 (1.5)
Cancer	5 (1.3)	25 (3.1)	30 (2.5)
Hepatic diseases	5 (1.3)	6 (0.8)	11 (0.9)
Autoimmunity diseases	5 (1.3)	15 (1.9)	20 (1.7)
Respiration rate, median, [IQR]	18 [16 – 20]	21.5 [19 – 25]	21 [18 – 25]
Medications in use, n (%)			
Hydroxychloroquine	6 (1.5)	99 (12.4)	105 (8.8)
Antibiotics	55 (13.8)	662 (82.9)	717 (59.8)
IL-6 mAbs	0 (0)	2 (0.3)	2 (0.2)
Ivermectin	23 (5.8)	33 (4.1)	56 (4.7)
Corticosteroids	14 (3.5)	316 (39.5)	330 (27.5)
Oseltamivir	0 (0)	255 (31.9)	255 (21.3)

IL-6 mAb: IL-6 monoclonal antibody; COPD: chronic obstructive pulmonary disease; IQR: interquartile range.

### Predictors of hospitalization

Out of 799 hospitalized patients, 39 (4.9%) had neurological complications, 194 (24.3%) had cardiovascular disturbances, 218 (27.3%) presented renal failure, and 241 (30.2%) presented secondary infections (Table 1S, Supplementary Material) during the hospitalization period.

**Figure 2:**
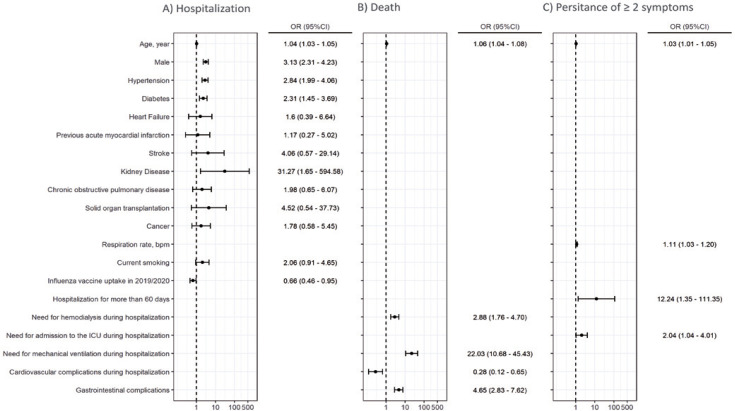
A) Logistic regression to estimate the risk of hospitalization due to COVID-19; B) Logistic regression to estimate the risk of death after hospitalization due to COVID-19; C) Logistic regression to estimate the risk of ≥2 symptoms persistence at 60 days in outpatient setting Bpm: breath per minute; 95%CI: 95% confidential interval; OR: odds ratio.

The previous influenza vaccination was linked to a reduced need for hospitalization following a COVID-19 diagnosis (Odds ratio [OR], 0.66; 95% confidence interval [95%CI], 0.46 to 0.95) (Figure 2A). However, the presence of chronic clinical conditions (hypertension, diabetes, and kidney disease) was associated with hospitalization (Table 2S, Supplementary Material).

### Predictors of mortality

A total of 292 patients died during hospitalization. Respiratory failure and shock secondary to COVID-19 were the principal causes of mortality (Table 3S, Supplementary Material).

Figure 2B reflects variables associated with mortality: age (OR=1.06; 95%CI=1.04 to 1.08), the need for hemodialysis (OR=2.88; 95%CI=1.76 to 4.70), the need for mechanical ventilation (OR=22.03; 95%CI=10.68 to 45.43), and cardiovascular (OR=4.65; 95%CI=2.83 to 7.62) and neurological (OR=0.28; 95%CI=0.12 to 0.65) clinical complications during hospitalization (Table 4S, Supplementary Material). Three patients in the hospitalized group died during the telemedicine follow-up.

### Predictors and persistence of symptoms in an outpatient setting

**Figure 3:**
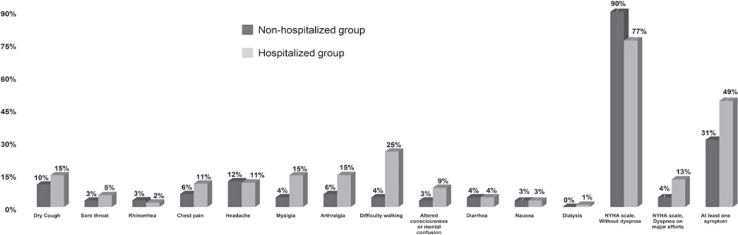
Symptoms reported among COVID-19 outpatients after 60 days NYHA dyspnea, New York Heart Association dyspnea scale: Dark Gray bars represent the non-hospitalized group, and gray bars represent the hospitalized group.

Dry cough, chest pain, headache, arthralgia, and myalgia were the most common and persistent symptoms during the 60 days, particularly in hospitalized patients. Among the hospitalized patients, 21% had persistent fatigue or tiredness at rest, and 25.4% had difficulty walking for 60 days (Table 5S, Supplementary Material). Hospitalized patients reported that altered consciousness or mental confusion worsened during follow-up in 13 (6.2%) and 18 (8.8%) patients at 15 and 60 days, respectively (Figure 3).

The persistence of more than two symptoms in 60 days was observed in 31.9 percent of patients (16% and 37.1% for the non-hospitalized and hospitalized groups, respectively). This was more prevalent in the patients with previous hospitalization histories (Table 6S, Supplementary Material). Variables associated with the persistence of more than two symptoms after 60 days of outpatient care included the following: an increase in age (OR=1.03; 95%CI= 1.01 to 1.05), respiratory rate at hospital admission (OR=1.11; 95%CI=1.03 to 1.20), more than 60 days of hospitalization (OR=12.24; 95%CI=1.35 to 111.35), and the need for admission to the intensive care unit (ICU) (OR=2.04; 95%CI=1.04 to 4.01) (Figure 3C).

## DISCUSSION

In the SARS-Brazil study, COVID-19 survivors who were older, tachypneic at admission, with hospital length of stay (LOS) > 60 days, and admitted to ICU had more persistent symptoms than COVID-19 patients who did not need hospitalization in this Brazilian cohort in early COVID-19 waves. In addition, higher mortality was observed during hospitalization (36% *versus* only one patient in the non-hospitalized group), particularly in aged patients needing hemodialysis and mechanical ventilation.

A recent Brazilian cohort study reported similar mortality rates and COVID-19 severity outcomes to our findings. However, the inpatient outcomes were COVID-19 waves and age dependence, which may be related to vaccination campaigns in Brazil.^(7)^

Regarding symptom persistence in an outpatient setting, our study showed that in 31.9% of cases, more than two symptoms were reported within two months. Similar data were found in a Swiss cohort study that included 629 patients, of which only 12.6 to 18.4 percent reported more than two symptoms seven months after infection.^(8)^ In this study, survivors of hospitalization reflected a higher prevalence of mental and cognitive alterations in 60 days in an outpatient setting compared to non-hospitalized patients.^(8)^

Recent reports have shown that a mentally altered status is associated with a prolonged LOS^(9,10)^ and may be the most frequent clinical manifestation of long-term COVID-19-related symptoms.^(11,12)^ Alternatively, our findings showed that previous influenza vaccination was associated with a low risk of hospitalization. However, apparent protection against severe COVID-19 could have arisen due to bias in cohort studies and may not translate into a biological cause-effect relationship.^(13-16)^ The controversial issues require further investigation.

Data from the survivors of the COALITION trials (COALITION VII),^(17)^ a Brazilian alliance of studies for the treatment and prevention of COVID-19, revealed that patients with more severe COVID-19 who required admission to the ICU during hospitalization had lower health-related quality-of-life scores, higher mortality, re-hospitalization, and new functional disabilities in the outpatient setting. Our findings reinforce that post-hospitalization COVID-19 patients had a clinically meaningful increase in functional disability persistence.

### Strengths and limitations

The strengths of this study include its prospective design, large sample size of the first COVID-19 patients in Brazil, and the assessment of patient-centered outcomes. Our study could explain the differences between hospitalized and non-hospitalized survivors’ outpatient settings. Therefore, our results could potentially be influenced by the COVID-19 vaccination. However, our study was conducted during a previous COVID-19 vaccine disposal in Brazil. This study had several limitations. First, the number of missing outpatient outcome assessments was relevant. In this case, selection bias is possible, as not all invitees participated. A high number of hospitalized individuals declined participation, resulting in the underrepresentation of these participants. We performed an analysis to limit the influence of selection bias on our results by weighing the participants back into invitees based on key baseline characteristics related to previous hospitalizations or outpatients. Second, the participants’ symptoms related to COVID-19 may have been associated with other conditions that may have occurred in the weeks after diagnosis or were potentially caused by SARS-CoV-2 infection.^(18,19)^ Third, we did not use a validated questionnaire for the signs and symptoms captured during telemedicine consultations. Potential limitations include the subjective interpretation of symptoms after COVID-19, which may have been affected by the telemedicine visits conducted during the follow-up period. This self-assessment approach can be used to determine whether symptoms result only from the SARS-CoV-2 infection mechanism or from the presence of comorbidities. In this context, clinical data on quality of life were not collected during telemedicine visits. We considered these data important to report in order to raise awareness of patients who suffer from long-COVID, especially hospitalized individuals. Therefore, in telemedicine visits, the small sample size that completed the follow-up can also be attributed to the higher mortality of hospitalized individuals, which might be due to disparities in access to telemedicine consultations. Finally, we did not include a Control Group of patients not SARS-CoV-2 infected to compare specific COVID-19 symptoms and critical illness effects on long-term outcomes.^(20-22)^

## CONCLUSION

Our data from the first COVID-19 wave in Brazil reflected a higher mortality rate, and COVID-19 survivors who were older, tachypneic at admission, with a length of stay >60 days, and admitted to the intensive care unit had more persistent symptoms than those who did not need hospitalization.

## SUPPLEMENTARY MATERIAL

Mortality, hospitalizations, and persistence of symptoms in the outpatient setting of the first COVID-19 wave in Brazil: results of SARS-Brazil cohort study


