# Supplementary material for: Mortality, hospitalizations, and persistence of symptoms in the outpatient setting of the first COVID-19 wave in Brazil: results of SARS-Brazil cohort study
Source: Einstein (Sao Paulo). 2024 Jul 12;22:eAO0652. doi: 10.31744/einstein_journal/2024AO0652 (PMC11323834; doi:10.31744/einstein_journal/2024AO0652)
Supplement: Supplementary file 1 [file 2317-6385-eins-22-eAO0652-suppl01.pdf]

SUPPLEMENTARY MATERIAL

Mortality, hospitalizations, and persistence of symptoms in the outpatient setting of the first COVID-19 wave in Brazil: results of SARS-Brazil cohort study

Henrique Andrade Rodrigues Fonseca, Adriano Jose Pereira, Ricardo Kenji Nawa, Viviane Aparecida Rodrigues Sant'Anna, Tatiana Ferreira de Almeida, Hélio Penna Guimarães, Alexandre Pereira Tognon, Lucas Miranda Marques, Lucas Santana Coelho da Silva, Rafaela de Souza Bittencourt, Camila Pachêco Gomes, Priscila de Aquino Martins, Aryadne Lyrio de Oliveira, Eveline Pipolo Milan, Frederico Toledo Campos Dall'Orto, Conrado Roberto Hoffman Filho, Guacyra Almeida, Fábio Barlem Hohmann, Diogo Duarte Fagundes Moia, Luciana Pereira Almeida Piano, Felipe Pinheiro Machado, Ronaldo Vicente Pereira Soares, Lucas Petri Damiani, Silvia Regina Lamas Assis, Edson Amaro Junior, Luiz Vicente Rizzo, Otávio Berwanger; on behalf of the SARS-BRAZIL Study Team

DOI: 10.31744/einstein_journal/2024AO0652

Executive Committee

Otávio Berwanger, Chair; Henrique Andrade Rodrigues Fonseca, Principal Investigator; Hélio Penna Guimarães; Tatiana Ferreira de Almeida; Edson Amaro Junior; Adriano Jose Pereira

Telemedicine Coordination

Adriano Jose Pereira and Ricardo Kenji Nawa

SARS-BRAZIL Study Team

Mônica Baumgardt Bay, Renata Bezerra Onofre, João Mauricio Dino do Nascimento, Renan Oliveira de Carvalho, Marianne Garcia de Oliveira, Julienne Karen Pacheco da Silva, Flavia Araújo da Silva, Maysa Mayran Chaves Moreira Bezerra - Hospital Giselda Trigueiro, Natal, RN, Brazil; Vinicius Santana Nunes - Hospital Estadual Dr. Jayme Santos Neves, Serra, ES, Brazil; Andressa Daron Giordani, Flávia Ghizzoni, Edson Junior Weber - Hospital São Vicente de Paulo, Passo Fundo, RS, Brazil; Ricardo Reinaldo Bergo, Gislayne Rogante Ribeiro, Elton Adriano Bonifacio, Ed Wilson de Jesus Carvalho Neves - Hospital Maternidade e Pronto Socorro Santa Lucia, Poços de Caldas, MG, Brazil; Drieli Meerholz - Hospital Hans Dieter Schmidt, Joinville, SC, Brazil; Guilherme Barreto Campos, Hellen Braga Martins Oliveira, Anna Carolina Saúde Dantas, Carolline Florentino Almeida - Universidade Federal da Bahia, Vitória da Conquista, BA, Brazil; Meton Soares de Alencar Filho, Jussara Alencar Arraes - Hospital e Maternidade São Vicente de Paulo, Barbalha, CE, Brazil; Mauro Esteves Hernandes - Santa Casa de Misericórdia de Votuporanga, Votuporanga, SP, Brazil; Evânio da Silva, Igor Barbosa Oliveira, Lívia Maria da Silva Gomes, Marília Barbosa de Albuquerque, Naydene Santos - Hospital de Emergência Dr. Daniel Houly, Arapiraca, AL, Brazil; Guilherme Neto, Bruna Azevedo - Hospital São Francisco de Assis, Belo Horizonte, MG, Brazil; Rodrigo Santana Dutra, Felipe Galdino Campos, Paloma Cristina Silva Pontes - Hospital Universitário Ciências Médicas/Fundação Educacional Lucas Machado, Belo Horizonte, MG, Brazil.

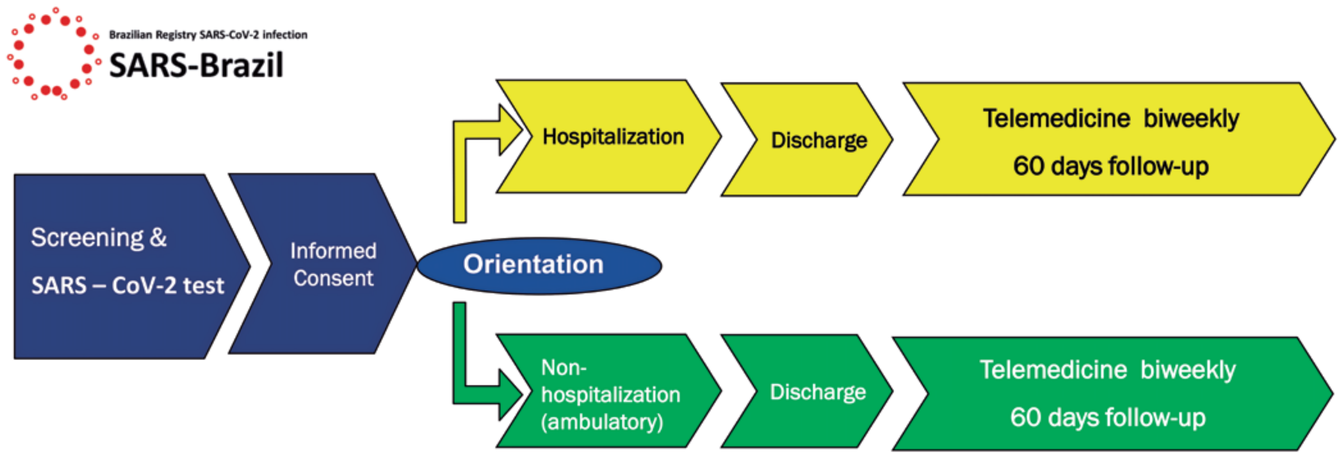

Figure 1S. Registry scheme for screening, SARS-CoV-2 nasopharynges test, indication for hospitalization or ambulatory care, and telemedicine 60days follow-up

Table 1S. Outcomes at hospital among hospitalized COVID-19 patients

Outcomes	Hospitalized Group n=799
Neurological complications, n (%)	39 (4.9)
Encephalopathy*	16 (41)
Epileptic attack*	9 (23.1)
Involvement of the peripheral nervous system*	6 (15.4)
Cardiovascular Complications, n (%)	194 (24.3)
QTc interval prolongation*	12 (6.2)
Clinically relevant ventricular arrhythmias*	42 (21.6)
Myocarditis*	5 (2.6)
Acute myocardial infarction*	6 (3.1)
Resuscitated cardiac arrest*	43 (22.2)
Cardiogenic shock*	131 (67.5)
Gastrointestinal events, n (%)	11 (1.4)
Renal failure / Need for hemodialysis, n (%)	218 (27.3)
Secondary infection, n (%)	241 (30.2)

* Event (%) subclassification of neurological or cardiological complications.

Table 2S. Univariate and multivariable analysis of clinical variables COVID-19-associate to hospitalization

Variables	Non-hospitalized Group (n=399)	Hospitalized Group (n=799)	Overall (n=1,198)	Univariate analysis p value	Multivariate analysis (n=1,106)*	
					OR (95%CI)	p value
Age, y, median, [IQR]	40 [32-53.5]	60 [49-70]	54 [41-67]	<0.001	1.04 (1.03-1.05)	<0.001
Male, n (%)	131 (32.8)	476 (59.6)	607 (50.7)	<0.001	3.13 (2.31-4.23)	<0.001
Chronic conditions, n (%)						
Hypertension	71 (17.8)	467 (58.4)	538 (44.9)	<0.001	2.84 (1.99-4.06)	<0.001
Diabetes	29 (7.3)	280 (35)	309 (25.8)	<0.001	2.31 (1.45-3.69)	<0.001
Heart failure	2 (0.5)	48 (6)	50 (4.2)	<0.001	1.6 (0.39-6.64)	0.51
Previous acute myocardial infarction	2 (0.5)	36 (4.5)	38 (3.2)	<0.001	1.17 (0.27-5.02)	0.83
Stroke	1 (0.3)	29 (3.6)	30 (2.5)	<0.001	4.06 (0.57-29.14)	0.16
Kidney disease	0 (0)	67 (8.4)	67 (5.6)	<0.001	31.27 (1.65-594.58)	0.022
Asthma	26 (6.5)	53 (6.6)	79 (6.6)	0.94	-	-
Chronic obstructive pulmonary disease	5 (1.3)	42 (5.3)	47 (3.9)	<0.001	1.98 (0.65-6.07)	0.23
Solid organ transplantation	1 (0.3)	17 (2.1)	18 (1.5)	0.012	4.52 (0.54-37.73)	0.16
Cancer	5 (1.3)	25 (3.1)	30 (2.5)	0.05	1.78 (0.58-5.45)	0.31
Hepatic diseases	5 (1.3)	6 (1.7)	11 (1.5)	0.61	-	-
Autoimmunity diseases	5 (1.3)	15 (1.9)	20 (1.7)	0.43	-	-
Respiration rate, median, [IQR]	18 [16-20]	21.5 [19-25]	21 [18-25]	<0.001	-	-
Current smoking, n (%)	10 (2.5)	65 (8.1)	75 (6.3)	<0.001	2.06 (0.91-4.65)	0.08
Influenza vaccine uptake in 2019/2020, n (%)	110 (27.6)	135 (19)	245 (22.1)	<0.001	0.66 (0.46-0.95)	0.023
Hydroxychloroquine, n (%)	6 (1.5)	99 (12.4)	105 (8.8)	<0.001	-	-
Antibiotics, n (%)	55 (13.8)	662 (83)	717 (59.9)	<0.001	-	-
IL-6 mAbs, n (%)	0 (0)	2 (0.6)	2 (0.3)	-	-	-
Ivermectin, n (%)	23 (5.8)	33 (9.4)	56 (7.5)	0.06	-	-
Corticosteroids, n (%)	14 (3.5)	316 (39.6)	330 (27.6)	<0.001	-	-
Oseltamivir, n (%)	0 (0)	255 (32)	255 (21.3)	<0.001	-	-

*Considering 10 significant variables in the univariate analysis and at least 90 valid responses, medications were excluded at baseline. Forward logistic regression was used to identify the significant predictors.
OR: odds ratio; IQR: interquartile range; IL-6 mAbs: interleukin 6 monoclonal antibodies; ICU: intensive care unit.

Table 3S. COVID-19-patients dead classification

Dead classification	n=292 (24.4%)*
Undefined, n (%)	1 (0.1)
Respiratory failure secondary to SARS-CoV-2 infection, n (%)	112 (9.3)
Septic shock secondary to SARS-CoV-2 infection, n (%)	62 (5.2)
Cardiogenic shock secondary to SARS-CoV-2 infection, n (%)	15 (1.3)
Shock from other etiologies (including mixed) secondary to SARS-CoV-2, n (%)	25 (2.1)
Renal failure, n (%)	8 (0.7)
Secondary infection, n (%)	8 (0.7)
Thromboembolic event (AMI, Stroke, Pulmonary embolism), n (%)	4 (0.3)
Others, n (%)**	57 (4.8)
Cytotoxic cerebral edema	1 (1.8)
Ischemic encephalopathy	1 (1.8)
Cardiac arrest	1 (1.8)
Viral pneumonia	1 (1.8)
Bacteria pneumonia	1 (1.8)
Other pneumonia	1 (1.8)
Respiratory insufficiency at home	1 (1.8)
No definitive cause	50 (87.7)

* For all patients (1,198); ** Others causes of death.

Table 4S. Univariate and multivariable analysis of clinical variables COVID-19-associate to mortality

Variables	Non-death (n=906)	Death (n=292)	Overall (n=1,198)	Univariate analysis p value	Multivariate analysis (n=1,120)*	
					OR (95%CI)	p value
Baseline						
Age, years, median, [IQR]	49 [37-62]	67 [58-76]	54 [41-67]	<0.001	1.06 (1.04-1.08)	<0.001
Male, n,	426 (47)	181 (62)	607 (50.7)	<0.001	-	-
Chronic conditions, n (%)						
Hypertension	340 (37.5)	198 (67.8)	538 (44.9)	<0.001	-	-
Diabetes	184 (20.3)	125 (42.8)	309 (25.8)	<0.001	-	-
Heart failure	26 (2.9)	24 (8.2)	50 (4.2)	<0.001	-	-
Acute myocardial infarction	19 (2.1)	19 (6.5)	38 (3.2)	<0.001	-	-
Stroke	12 (1.3)	18 (6.2)	30 (2.5)	<0.001	-	-
Kidney disease	26 (2.9)	41 (14)	67 (5.6)	<0.001	-	-
Asthma	67 (7.4)	12 (4.1)	79 (6.6)	0.049	-	-
Chronic obstructive pulmonary disease	26 (2.9)	21 (7.2)	47 (3.9)	<0.001	-	-
Solid organ transplantation	12 (1.3)	6 (2.1)	18 (1.5)	0.41	-	-
Cancer	18 (2)	12 (4.1)	30 (2.5)	0.043	-	-
Hepatic diseases	9 (1.4)	2 (1.8)	11 (1.5)	0.67	-	-
Autoimmunity diseases	16 (1.8)	4 (1.4)	20 (1.7)	0.79	-	-
Respiration rate, bpm, median, IQR	20 [18-24]	22 [20-26]	21 [18-25]	<0.001	-	-
Current smoking, n (%)	44 (4.9)	31 (10.6)	75 (6.3)	<0.001	-	-
Influenza vaccine uptake in 2019/ 2020, n (%)	226 (25.7)	19 (8.4)	245 (22.1)	<0.001	-	-
Medications in use previous hospitalization, n (%)						
Hydroxychloroquine	66 (7.3)	39 (13.4)	105 (8.8)	0.001	-	-
Antibiotics	458 (50.6)	259 (88.7)	717 (59.9)	<0.001	-	-
IL-6 mAbs	0 (0)	2 (1.8)	2 (0.3)	-	-	-
Ivermectin	53 (8.3)	3 (2.7)	56 (7.5)	0.039	-	-
Corticosteroids	201 (22.2)	129 (44.2)	330 (27.6)	<0.001	-	-
Oseltamivir	136 (15)	119 (40.8)	255 (21.3)	<0.001	-	-
During hospitalization, n (%)						
In use of dobutamine	6 (1.7)	10 (4.7)	16 (2.8)	0.033	-	-
In use of dopamine	5 (1.4)	3 (1.4)	8 (1.4)	1.00	-	-
In use of noradrenaline	90 (25)	149 (69.3)	239 (41.6)	<0.001	-	-
In use of adrenaline	1 (0.3)	5 (2.3)	6 (1)	0.029	-	-
In use of vasopressin	4 (1.1)	29 (13.5)	33 (5.7)	<0.001	-	-
Hospitalization for more than 60 days	7 (1.1)	9 (8.4)	16 (2.2)	<0.001	-	-
Need for hemodialysis during hospitalization	61 (6.7)	157 (54.3)	218 (18.3)	<0.001	2.88 (1.76-4.70)	<0.001
Need for admission to the ICU during hospitalization	359 (42.5)	268 (95.4)	627 (55.7)	<0.001	-	-
Need for mechanical ventilation during hospitalization	182 (21.7)	276 (95.8)	458 (40.7)	<0.001	22.03 (10.68-45.43)	<0.001
Need for non-invasive ventilation during hospitalization	157 (18.7)	56 (20.1)	213 (19.1)	0.60	-	-
Secondary infection during hospitalization	99 (11)	142 (49)	241 (20.2)	<0.001	-	-
Neurological complications during hospitalization	21 (2.3)	18 (6.3)	39 (3.3)	0.001	0.28 (0.12-0.65)	0.003
Cardiovascular complications during hospitalization	50 (5.5)	144 (49.8)	194 (16.3)	<0.001	4.65 (2.83-7.62)	<0.001
Gastrointestinal complications	6 (0.9)	5 (4.5)	11 (1.5)	0.014	-	-

*Considering the significant variables at 10% in the univariate analysis and with at least 90% of valid responses, medications in baseline. Forward logistic regression was used to identify the significant predictors.
OR: odds ratio; IQR: interquartile range; IL-6 mAbs: interleukin 6 monoclonal antibodies; ICU: Intensive care unit.

Table 5S. Outcomes among COVID-19 patients in telemedicine visits

Outcomes	Non-hospitalized Group n=71[£]	Hospitalized Group n=210[£]	Non-hospitalized n=69	Hospitalized n=206	Non-hospitalized n=68	Hospitalized n=206	Non-hospitalized n=68	Hospitalized n=205
Died during hospitalization or discharge previous telemedicine admission, n (%)*	1 / 399 (0.3)	288 / 799 (36.0)	-	-	-	-	-	-
	15-day telemedicine care		30-day telemedicine care		45-day telemedicine care		60-day telemedicine care	
Need ambulatory visit, n (%)	3 (4.2)	14 (6.7)	1 (1.4)	6 (2.9)	6 (8.8)	5 (2.4)	0 (0)	3 (1.5)
Re-hospitalizations, n (%)	0 (0)	7 (3.3)	0 (0)	3 (1.5)	1 (1.5)	1 (0.5)	0 (0)	0 (0)
Oxygen supplementation	1 (1.4)	10 (4.8)	0 (0)	2 (1)	1 (1.5)	2 (1)	0 (0)	1 (0.5)
ICU admission	0 (0)	3 (1.4)	0 (0)	2 (1)	0 (0)	1 (0.5)	0 (0)	0 (0)
Invasive ventilation	0 (0)	0 (0)	0 (0)	0 (0)	0 (0)	0 (0)	0 (0)	0 (0)
Signs and symptoms of COVID-19, n (%)								
Dry cough	25 (35.2)	63 (30)	7 (10.1)	51 (24.9)	8 (11.8)	33 (16)	7 (10.3)	30 (14.6)
Cough with phlegm	5 (7)	16 (7.7)	2 (2.9)	7 (3.4)	2 (2.9)	10 (4.9)	1 (1.5)	4 (2)
Sore throat	9 (12.7)	25 (12)	2 (2.9)	10 (4.9)	4 (5.9)	15 (7.3)	2 (2.9)	11 (5.4)
Rhinorrhea	8 (11.3)	6 (2.9)	4 (5.9)	4 (1.9)	3 (4.4)	3 (1.5)	2 (3)	4 (2)
Chest pain	7 (9.9)	34 (16.2)	10 (14.5)	28 (13.6)	8 (11.8)	22 (10.7)	4 (5.9)	22 (10.7)
Headache	30 (42.3)	45 (21.4)	19 (27.5)	39 (18.9)	15 (22.1)	28 (13.6)	8 (11.8)	23 (11.2)
Myalgia	19 (26.8)	38 (18.2)	10 (14.5)	30 (14.6)	4 (5.9)	31 (15)	3 (4.4)	30 (14.6)
Arthralgia	9 (12.7)	33 (15.7)	5 (7.2)	35 (17)	8 (11.8)	29 (14.1)	4 (5.9)	30 (14.7)
Fatigue or tiredness at rest	27 (38)	83 (39.5)	15 (21.7)	64 (31.1)	9 (13.2)	55 (26.8)	4 (5.9)	43 (21)
Difficulty walking, n (%)	10 (14.1)	79 (37.8)	5 (7.2)	71 (34.6)	8 (11.8)	65 (31.9)	3 (4.4)	52 (25.4)
Altered consciousness or mental confusion	3 (4.2)	13 (6.2)	0 (0)	5 (2.4)	2 (2.9)	13 (6.3)	2 (2.9)	18 (8.8)
Abdominal pain,	3 (4.2)	5 (2.4)	3 (4.3)	5 (2.5)	1 (1.5)	4 (1.9)	1 (1.5)	2 (1)
Diarrhea	12 (16.9)	22 (10.5)	4 (5.8)	6 (2.9)	1 (1.5)	4 (1.9)	3 (4.4)	9 (4.4)
Nausea	10 (14.1)	14 (6.7)	2 (2.9)	7 (3.4)	3 (4.4)	4 (1.9)	2 (2.9)	6 (2.9)
Vomiting	3 (4.2)	7 (3.3)	2 (2.9)	2 (1)	1 (1.5)	3 (1.5)	0 (0)	1 (0.5)
Other infections	1 (1.4)	2 (1)	0 (0)	0 (0)	0 (0)	1 (0.5)	0 (0)	0 (0)
Dialysis	0 (0)	2 (1)	0 (0)	2 (1)	0 (0)	2 (1)	0 (0)	2 (1)
Bleeding	1 (1.4)	1 (0.5)	0 (0)	1 (0.5)	0 (0)	0 (0)	0 (0)	0 (0)
NYHA Dyspnea scale, n (%)								
Without dyspnea	62 (87.3)	187 (89)	50 (73.5)	130 (63.7)	56 (82.4)	137 (66.5)	61 (89.7)	157 (76.6)
Dyspnea on major efforts	9 (12.7)	23 (11)	6 (8.8)	34 (16.7)	7 (10.3)	44 (21.4)	3 (4.4)	26 (12.7)
Dyspnea on moderate efforts	0 (0)	0 (0)	10 (14.7)	37 (18.1)	3 (4.4)	22 (10.7)	3 (4.4)	21 (10.2)
Dyspnea at rest	0 (0)	0 (0)	2 (2.9)	3 (1.5)	2 (2.9)	3 (1.5)	1 (1.5)	1 (0.5)
Patients at last 1 and ≥2 symptoms, n (%)								
At least one symptom	51 (71.8)	149 (71)	35 (50.7)	131 (63.6)	32 (47.1)	114 (55.3)	21 (30.9)	100 (48.8)
At least 2 symptoms	43 (60.6)	115 (54.8)	21 (30.4)	94 (45.6)	22 (32.4)	79 (38.3)	11 (16.2)	76 (37.1)
Death in telemedicine visits	0 (0)	2 (1)	0 (0)	0 (0)	0 (0)	0 (0)	0 (0)	1 (0.5)

[£] Outpatient setting; * All included patients. The groups were hospitalized or non-hospitalized prior to the telemedicine visits.

Table 6S. Univariate and multivariable analysis of clinical variables COVID-19-associate to persistence ≥2 symptoms in the outpatient setting of 60 days

Variables	≥2 symptoms (n=87)	<2 symptoms (n=186)	Overall (n=273)	Univariate analysis p value	Multivariate analysis (n=194)*	
					OR (95%CI)	p value
Baseline						
Age, *y*, median, [IQR]	57 [47 – 67]	45 [35 – 62]	53 [38.5-63.5]	<0.001	1.03 (1.01-1.05)	0.015
Male, n (%)	39 (44.8)	94 (50.5)	133 (48.7)	0.380	-	-
Chronic conditions, n (%)						
Hypertension	47 (54)	67 (36)	114 (41.8)	0.005	-	-
Diabetes	27 (31)	34 (18.3)	61 (22.3)	0.018	-	-
Heart failure	5 (5.7)	7 (3.8)	12 (4.4)	0.530	-	-
Previous acute myocardial infarction	5 (5.7)	2 (1.1)	7 (2.6)	0.035	-	-
Stroke	1 (1.1)	4 (2.2)	5 (1.8)	1.000	-	-
Kidney Disease	4 (4.6)	5 (2.7)	9 (3.3)	0.472	-	-
Asthma	10 (11.5)	11 (5.9)	21 (7.7)	0.107	-	-
Chronic obstructive pulmonary disease	3 (3.4)	4 (2.2)	7 (2.6)	0.683	-	-
Solid organ transplantation	3 (3.4)	2 (1.1)	5 (1.8)	0.331	-	-
Cancer	3 (3.4)	2 (1.1)	5 (1.8)	0.331	-	-
Hepatic diseases	2 (2.3)	5 (2.7)	7 (2.6)	1.000	-	-
Autoimmunity diseases	4 (4.6)	4 (2.2)	8 (2.9)	0.270	-	-
Respiratory rate, bpm, median, [IQR]	20 [18 – 23]	19 [18-21]	19.5 [18 – 22]	0.003	1.11 (1.03-1.20)	0.005
Current smoking, n (%)	1 (1.1)	3 (1.6)	4 (1.5)	1.000	-	-
Influenza vaccine uptake in 2019/2020, n (%)	33 (44.6)	88 (49.7)	121 (48.2)	0.459	-	-
Medications in use previous hospitalization, n (%)						
Hydroxychloroquine	4 (4.7)	6 (3.2)	10 (3.7)	0.730	-	-
Antibiotics	71 (82.6)	109 (58.6)	180 (66.2)	<0.001	-	-
IL-6 mAbs	0 (0)	0 (0)	0 (0)	-	-	-
Ivermectin	11 (12.8)	31 (16.7)	42 (15.4)	0.411	-	-
Corticosteroids	46 (53.5)	80 (43)	126 (46.3)	0.107	-	-
Oseltamivir	8 (9.3)	12 (6.5)	20 (7.4)	0.402	-	-
Previous hospitalization	76 (88.4)	129 (69.4)	205 (75.4)	0.001	-	-
During hospitalization, n (%)						
In use of dobutamine	0 (0)	0 (0)	0 (0)	-	-	-
In use of dopamine	0 (0)	1 (1.9)	1 (1.1)	-	-	-
In use of noradrenaline	2 (5.1)	0 (0)	2 (2.2)	-	-	-
In use of adrenaline	0 (0)	0 (0)	0 (0)	-	-	-
In use of vasopressin	0 (0)	0 (0)	0 (0)	-	-	-
Hospitalization for more than 60 days	6 (7)	1 (0.6)	7 (2.6)	0.005	12.24 (1.35-111.35)	0.026
Need for haemodialysis during hospitalization	3 (3.4)	2 (1.1)	5 (1.8)	0.331	-	-
Need for admission to the ICU during hospitalization	55 (63.2)	66 (35.5)	121 (44.3)	<0.001	2.04 (1.04-4.01)	0.038
Need for mechanical ventilation during hospitalization	11 (12.9)	11 (5.9)	22 (8.1)	0.049	-	-
Need for non-invasive ventilation during hospitalization	34 (40)	41 (22)	75 (27.7)	0.002	-	-
Secondary infection during hospitalization	10 (11.5)	10 (5.4)	20 (7.3)	0.070	-	-
Neurological complications during hospitalization	2 (2.3)	0 (0)	2 (0.7)	-	-	-
Cardiovascular complications during hospitalization	4 (4.6)	2 (1.1)	6 (2.2)	0.084	-	-
Gastrointestinal complications	2 (2.3)	2 (1.1)	4 (1.5)	0.594	-	-

*Model with variable selection, considering all significant variables (p<0.10) in the univariate analysis (except medications) and with at least 90% of information completed. Forward logistic regression was used to identify the significant predictors.
OR: odds ratio; IQR: interquartile range; 95%CI: 95% confidence interval; IL-6 mAbs: interleukin 6 monoclonal antibodies; ICU: intensive care unit.
